# Epistemic beliefs’ role in promoting misperceptions and conspiracist ideation

**DOI:** 10.1371/journal.pone.0184733

**Published:** 2017-09-18

**Authors:** R. Kelly Garrett, Brian E. Weeks

**Affiliations:** 1 School of Communication, Ohio State University, Columbus, Ohio, United States of America; 2 Department of Communication Studies and Center for Political Studies, University of Michigan, Ann Arbor, Michigan, United States of America; Institut Català de Paleoecologia Humana i Evolució Social (IPHES), SPAIN

## Abstract

Widespread misperceptions undermine citizens’ decision-making ability. Conclusions based on falsehoods and conspiracy theories are by definition flawed. This article demonstrates that individuals’ epistemic beliefs–beliefs about the nature of knowledge and how one comes to know–have important implications for perception accuracy. The present study uses a series of large, nationally representative surveys of the U.S. population to produce valid and reliable measures of three aspects of epistemic beliefs: reliance on intuition for factual beliefs (*Faith in Intuition for facts*), importance of consistency between empirical evidence and beliefs (*Need for evidence*), and conviction that “facts” are politically constructed (*Truth is political*). Analyses confirm that these factors complement established predictors of misperception, substantively increasing our ability to explain both individuals’ propensity to engage in conspiracist ideation, and their willingness to embrace falsehoods about high-profile scientific and political issues. Individuals who view reality as a political construct are significantly more likely to embrace falsehoods, whereas those who believe that their conclusions must hew to available evidence tend to hold more accurate beliefs. Confidence in the ability to intuitively recognize truth is a uniquely important predictor of conspiracist ideation. Results suggest that efforts to counter misperceptions may be helped by promoting epistemic beliefs emphasizing the importance of evidence, cautious use of feelings, and trust that rigorous assessment by knowledgeable specialists is an effective guard against political manipulation.

## Introduction

Misperceptions about the scientific and political world pose a fundamental threat to democracy, undermining citizens’ ability to make decisions that effectively promote both individual self-interest and the social good [1]. Over the past fifteen years, widespread endorsement of falsehoods has become a defining feature of the political landscape [2]. Large segments of the U.S. population have expressed the inaccurate belief that Iraq had weapons of mass destruction prior to the U.S.-led invasion [3], that President Obama was not born in the U.S. [4], that climate change is a hoax perpetrated to advance a political agenda [5], and many others. Collective decision making is premised on a common understanding of a shared reality; it is incompatible with the widespread rejection of rigorously assessed and widely available evidence.

Finding ways to reduce misperceptions is vital to the democratic endeavor, and researchers from across the social sciences are actively searching for solutions, but the problem is notoriously difficult [6, 7]. Identifying factors that contribute to misperceptions is critical because effective corrective messaging strategies are grounded in an understanding of the mechanisms by which misperceptions take hold [8]. A variety of psychological processes have been identified. Individuals have a propensity to maintain beliefs that are consistent with their political ideology, economic worldview, and moral values [9–11]. Beliefs serve a social purpose, and rejecting a claim endorsed by the in-group risks ostracism [12]. Exposure to ideologically oriented news media promote beliefs advantageous to the favored party [13]. Processing strategies that allow individuals to make decisions in the face of an immensely complex information environment can also lead people astray. For example, repeated exposure and ease of understanding are often taken as indicators of accuracy [14]; and individuals are powerfully attracted to causal explanations, and will continue to embrace them even after the evidence on which they were formed has been rejected [15].

There is also some evidence that individuals’ styles of thinking can influence their willingness to accept claims lacking empirical evidence. Individuals who tend to see intentional agency behind every event are more likely to believe conspiracy theories [16], as are those who attribute extraordinary events to unseen forces or interpret events through the Manichean narrative of good versus evil [17]. Those who mistrust authority, who are convinced that nothing is as it seems, and who lack control over their environment are also more predisposed to conspiracist ideation [18–21].

Individuals’ epistemic beliefs, however, remain an understudied topic in misperceptions research. Epistemic beliefs are beliefs about the nature of knowledge and how one comes to know, and they affect general comprehension, reason, and learning [22, 23]. They should have implications for the accuracy of individuals’ beliefs about the scientific and political world. We focus on three distinct epistemic beliefs, related to the roles that feelings, evidence, and politics each have in shaping what one knows. We chose these areas based on their prominence in related literatures. (1) *Feelings* are increasingly recognized as a source of insight in the decision-making literature [24, 25], but they are also prone to bias [14, 26]. (2) The value of *evidence* when forming beliefs may appear self-evident, but recent research has shown that at least some individuals are willing to embrace claims that contradict what they know of the evidence [27]. Finally, (3) controversy over the extent to which facts are *politically constructed* has moved from the academy into mainstream [2, 28]. Next, we conceptualize each of these epistemic belief.

## Epistemic beliefs

### Faith in Intuition for facts (FI-facts)

Individuals rely on two complementary processes when forming judgments, including judgments about what is true and what is not. Although scholars differ over the precise nature of these processes, there is agreement about their general contours. One process is rapid, automatic, and requires little conscious thought, whereas the other is slower, more deliberate, and more systematic [25, 29]. The former resembles intuition, whereas the latter more closely resembles reason or deliberation. The two processes are distinct and complementary. Although beliefs are commonly understood to be the product of reason–of weighing the evidence for and against a claim in order to reach a conclusion–there is evidence that intuition is integral to the process as well. Furthermore, there is evidence that bodily experiences and feelings associated with information processing are valuable, facilitating effective decision making [24, 29]. When faced with complex judgments, people often quickly and subconsciously ask themselves, “How do I feel about it?” The resulting feelings serve as a quick and intuitive heuristic that informs their decision [30]. Individuals who are unable to use their intuition, or “gut feelings,” are prone to making bad decisions even when their reasoning skills are high [24].

The shortcomings of intuition as a means of assessing the accuracy of a claim is captured by the notion of “truthiness”. The term, popularized by American satirist Stephen Colbert, refers to the subjective feeling that something is true, regardless of the evidence [31]. As satisfying and expedient as it may be to consider whether something “feels right” when assessing its accuracy, intuitive errors can occur for many reasons, including bias from existing attitudes or beliefs, or the misattribution of coincidentally co-occurring emotions [26, 30]. These errors can lead individuals to ignore or distrust important information, culminating in beliefs that contradict available evidence. In other words, habitually trusting one’s intuition or feelings may contribute to misperceptions. This is consistent with evidence that analytic thinking–the opposite of relying on instinct or intuition–tends to attenuate conspiracist ideation [32].

### Need for evidence

The second epistemic belief we examine concerns the value an individual places on ensuring that beliefs are consistent with available evidence. Whereas *FI-facts* pits intuition against logic and reason, the emphasis here is on the compatibility of beliefs with externally validated data. In other words, is confirmatory evidence essential to belief maintenance? Or, alternatively, can a belief persist in the face of contradictory evidence? A growing body of scholarship suggests that some individuals’ beliefs are only weakly correlated with their knowledge of relevant information. For example, knowledge about climate science is a poor predictor of conservatives’ belief in climate change [27]. Many on the political right know that climate scientists believe anthropogenic climate change is real, while simultaneously rejecting the conclusion themselves. Exposure to partisan news media on the left or right exacerbates this tendency, making it more likely that news consumers express inaccurate beliefs, even when they are aware of evidence to the contrary [33].

Given that accurate beliefs are here defined as those that best align with available evidence, *Need for evidence* should be uniformly associated with holding fewer misperceptions. Individuals who view evidence as playing an essential role in the belief formation process, and who more consistently reject claims that do not square with available data should be less likely to engage in conspiracist ideation, and less likely to accept politically expedient falsehoods.

### Truth is political

Facts do not have the authority they once did [2]. This is not to suggest that there was ever universal accord about what is true and what is not. It has long been understood that the boundaries between fact and interpretation are not always obvious [34], and that claims of absolute knowledge should be viewed with skepticism [35]. Today, however, the idea that truth is relative, and that facts are shaped by social and political processes, is widespread. Those who believe that truth is politically constructed hold that “facts” cannot be entirely separated from the political or social system from which they arise. In other words, what is considered fact is subjective and politically determined [36]. In the words of one prominent political scientist, “if a fact is worth thinking about in making a policy choice, it is probably worth disputing” [34, p148].

The idea that facts are socially constructed is not constrained to political topics: scientific truths are also increasingly understood to be shaped by social processes. This may be due in part to the turn toward social constructivism in the social sciences. Scholars have observed that scientific facts are constructed through social processes, and that they can be subject to pressures unrelated to scientific inquiry [37]. The Internet also may exacerbate problems associated with the social construction of facts by allowing non-experts to create and share content that challenges expert and scientific consensus, providing apparent legitimacy to multiple “truths,” and facilitating a post-modern paradigm in which there are no objective facts [38]. At its extreme, social constructivism is equated with the assertion that science is only one among many equally valid ways of knowing the world [39]. Although this line of scholarship was never intended to undermine the value of science for informing judgment and making policy decisions, it has been used toward this end [40, 41]. Indeed, science educators sometimes worry that social constructivism may cause students to doubt scientific certainty [42]. And some scientists bristle at these socially informed accounts, blaming them for the public’s growing distrust in science, and for individuals’ reliance on any belief system that challenges the primacy of science [28].

### Conditioning on ideology

Individuals are prone to believe misperceptions that are consistent with their political identity, and this bias increases with opportunity (e.g., time to think) and with ability (e.g., cognitive resources) [12, 43]. Faced with a politically unpalatable claim, individuals use the resources at their disposal to resist it. It is possible that epistemic beliefs also are deployed in order to defend existing beliefs. If so, then their effect will be conditioned on ideology. For example, it might be that *Need for evidence* will cause liberals (who have social motivations to accept the claim that climate change is real) to be more accurate, while simultaneously leading conservatives (who are socially motivated to reject climate science) to be less accurate. This same logic could apply to any of the three epistemic beliefs.

## Current research

We anticipate that the three epistemic beliefs, *FI-facts*, *Need for evidence*, and *Truth is political*, will function independently, each helping to shape the accuracy of an individual’s perceptions. Further, we conceptualize these epistemic beliefs as temporally stable, but not fixed. Changes in how one understands the nature of knowledge has profound implications for the belief formation process, which should lead individuals to resist frequent change. However, we would expect social factors, such as socialization and learning, to produce gradual shifts.

The objectives of this research are two-fold. First, we develop and validate a series of short scales corresponding to three epistemic beliefs. The scales are tested using surveys conducted with three separate, nationally representative samples. We use a structural equation model-based exploratory factor analysis (EFA) of data collected in 2015 to select items that load cleanly onto the three factors, and we perform confirmatory factor analysis (CFA) with two separate datasets collected during the 2016 U.S. Presidential election to validate the measurement model. Second, we assess whether these scales enhance our ability to estimate conspiracist ideation and/or belief accuracy on several issues. We estimate a series of latent regression models using the 2016 data, regressing respondents’ perceptions of reality on the epistemic belief scales and a variety of previously established predictors. Results demonstrate that epistemic beliefs have a substantively important influence on individuals’ (mis)perceptions.

This research was approved by the Ohio State University’s Institutional Review Board. Consent was given digitally, via an online survey tool.

## Study 1

### Methods

The first study involves generating candidate items, administering the items to a representative sample of Americans, conducting factor analysis, and selecting items for use in each of the three proposed scales.

#### Sample

We worked with GfK, a market research company, to field a survey using its KnowledgePanel, a probability-based web panel designed to be representative of the adult population of the United States. GfK uses address-based sampling methods to recruit the panel, which consists of general population adults age 18 and over. After consenting to participate in the panel, panelists were sent email notifications requesting they complete the survey. They received a small reward for participating. Data from this first internally funded survey (“2015 OSoC”) were collected between February 25 and March 9, 2015. The sample included 510 respondents (51.8% female; *mean* age = 46.8, *SD* = 17.5). Other demographics for participants were comparable to the U.S. population (see S1 Appendix for more detail). Given the high respondent-to-item ratio, this sample size is more than adequate for EFA [44].

#### Measures

The 2015 OSoC survey included a series of 20 candidate items intended to measure the three epistemic beliefs. (See S2 Appendix for a description of the item generation process and question wording for all items.)

## Results

We use structural equation modeling (SEM) to examine factor loadings among the twenty candidate measurement items [45]. Based on the initial EFA, several cross-loaded items were dropped, leaving four items for each of the three concepts. The resulting model achieves good model fit by conventional standards [46], *CFI* = .98, *RMSEA* = .04, *SRMR* = .02 (see Table 1 for selected items and their wording; see S1 Table for factor loadings).

**Table 1:** Epistemic beliefs item wording.

**Faith in Intuition for facts:**	
*Feel1*	I trust my gut to tell me what’s true and what’s not
*Feel2*	I trust my initial feelings about the facts
*Feel3*	My initial impressions are almost always right
*Feel4*	I can usually feel when a claim is true or false even if I can’t explain how I know
**Need for evidence:**	
*Evid1*	Evidence is more important than whether something feels true
*Evid2*	A hunch needs to be confirmed with data
*Evid3*	I trust the facts, not my instincts, to tell me what is true
*Evid4*	I need to be able to justify my beliefs with evidence
**Truth is political:**	
*Poli1*	Facts are dictated by those in power
*Poli2*	What counts as truth is defined by power
*Poli3*	Scientific conclusions are shaped by politics
*Poli4*	“Facts” depend on their political context

Response options range from “strongly disagree” to “strongly agree”, with “neither agree nor disagree” as the midpoint.

## Studies 2 & 3

### Methods

The next two studies use nationally representative survey data to assess the new scales. In both surveys, respondents reported their factual beliefs (either about conspiracy theories or high-profile issues) before being presented with the epistemic belief measures. In Study 3 several other questions were presented between the two sets of measures. Respondents also provided information about their demographic and psychological characteristics.

#### Sample

Like Study 1, these two studies also use data collected via GfK’s KnowledgePanel. Study 2 was part of a three-wave University-funded omnibus panel survey (“2016 OSoC”). Epistemic beliefs and conspiracist ideation were both measured in wave two, which was fielded between October 4-12, 2016 and included 630 respondents (76.4% retention rate from first wave; 51.7% female; *mean* age = 47.43, *SD* = 17.48). Study 3 was an NSF-sponsored three-wave panel survey in which respondents were recontacted three times over the course of the election (“2016 NSF”). The baseline survey was conducted between July 29 and August 11, 2016, and included 965 respondents (51.8% female; *mean* age = 46.93, *SD* = 17.60). 764 (79.2% retention rate) completed wave 2, which was fielded September 14-22, 2016. The third wave included 629 respondents (65.2% retention rate from baseline, 82.3% from wave 2) and was collected November 9-14. (Other sample demographics are reported in S1 Appendix) These samples are large enough to detect even small effects.

### Measures

#### Epistemic beliefs

Based on the results of the initial EFA from the 2015 OSoC survey, respondents in the 2016 OSoC survey were presented with 11 items measuring the three epistemic beliefs. (One item was inadvertently omitted by the survey company when administering the survey.) Items were measured on a nine-point scale, from strongly disagree (1) to strongly agree (9). The 2016 NSF survey included all 12 epistemic belief questions, measured on a five-point scale coded in the same direction. (See S2 Appendix for composite scale reliability statistics.)

#### Conspiracist ideation

Conspiracist ideation describes individuals’ willingness to endorse conspiracy theories–unwarranted explanations of social phenomena that cite as a primary cause a small group of powerful individuals acting in secret for their own benefit, often at the expense of the public good [16]. Following prior research [5], conspiracist ideation was measured using belief in several prominent conspiracy theories (see Table 2), with responses given on a nine-point scale anchored by definitely not true (1) and definitely true (9).

**Table 2:** Conspiracist ideation (OSoC 2016).

Statement	Endorsing conspiracy
The assassination of John F. Kennedy was not committed by the lone gunman Lee Harvey Oswald but was rather a detailed organized conspiracy to kill the President.	45.7%
The assassination of Martin Luther King Jr. was the result of an organized conspiracy by U.S. government agencies such as the CIA and FBI.	33.1
Princess Diana’s death was not an accident but rather an organized assassination by members of the British royal family who disliked her.	32.1
A powerful and secretive group known as the New World Order are planning to eventually rule the world through an autonomous world government which would replace sovereign governments.	30.7
The U.S. government allowed the 9–11 attacks to take place so that it would have an excuse to achieve foreign (e.g., wars in Afghanistan and Iraq) and domestic (e.g., attacks on civil liberties) goals that had been determined prior to the attacks.	23.9
U.S. agencies intentionally created the AIDS epidemic and administered it to Black and gay men in the 1970s.	22.8
The Apollo moon landings never happened and were staged in a Hollywood film studio.	15.3

Response options range from “definitely not true” (1) to “definitely true” (9). Scores higher than 5 are treated as an endorsement for this table.

#### Beliefs about issues

The 2016 NSF survey measured belief accuracy about four prominent claims related to political and scientific issues. Respondents were presented with pairs of contrasting statements and asked to place a mark on a five-point scale closer to the endpoint that best described their personal beliefs, placing the mark in the middle if they were unsure of the truth (see Table 3). Responses were recoded following data collection so that higher scores represent greater accuracy, resulting in a scale ranging from 1 (most inaccurate) to 5 (most accurate).

**Table 3:** Beliefs about issues (NSF 2016).

Statement	*M*(*SD*)
Human activity is contributing to changes in the global climate (5)—Human activity has no influence on global climate (1)	3.69 (1.23)
Most Muslims support violence against Western countries, including the U.S.(1)—Most Muslims oppose violence against Western countries, including the U.S. (5)	3.46 (1.23)
Iraq had weapons of mass destruction immediately before the Iraq war began (1)—Iraq had no weapons of mass destruction immediately before the Iraq war began (5)	3.13 (1.34)
Vaccines cause autism (1)—Vaccination is unrelated to autism (5)	3.75 (1.19)

Responses scored from 1 (most inaccurate) to 5 (most accurate), with a score of 3 indicating ambivalence or uncertainty about what is true.

#### Other measures

Some of the following analyses account for a variety of factors known to influence misperceptions, including religious fundamentalism, Need For Cognition, education, political party affiliation and ideology, attention to politics, and use of partisan news. A complete list of question wording and measure descriptives is included in S2 Appendix.

## Results

### Measurement model

A series of four SEM-based CFAs using both 2016 surveys confirm that the measurement model is robust. Model fit across all tests was good (Table 4; and see S1 Fig for sample factor loading) according to conventional standards (*CFI* ≥ .95 and *RMSEA* and *SRMR* ≤ .06 [46]). Scale reliability for composite scales was also acceptable in all four surveys, *alphas*_feel_ = .78–.80, *alphas*_evid_ = .81–.83, *alphas*_poli_ = .75–.81.

**Table 4:** Beliefs about issues (NSF 2016).

	*CFI*	*RMSEA*	*SMRM*
2016 OSoC ^a^	0.956	0.061	0.048
2016 NSF, Wave 1	0.963	0.050	0.045
2016 NSF, Wave 2	0.959	0.057	0.049
2016 NSF, Wave 3	0.960	0.052	0.050

^a.^ Based on a three-item measure of *Truth is political* (excluding Poli4)

#### Statistics and correlations

Composite scale descriptives and zero-order correlations for the three epistemic belief measures are reported in Table 5 (distributions for composite scales are presented visually in S2 Fig). The scales are only weakly correlated with factors often associated with misperceptions. Test-retest reliability in the 2016 NSF panel data was adequate. In the most rigorous test, comparing responses collected in August to those collected in November, the test-retest correlations were acceptable, *r*_feel_ = .58, *p* < .001; *r*_evid_ = .58, *p* < .001; *r*_poli_ = .56, *p* < .001. Although values above 0.7 are ideal, psychological tests frequently yield lower values [47]. Furthermore, the reliability reported here is consistent with our assertion that these attributes are temporally stable, but not fixed.

**Table 5:** Epistemological belief composite scores, descriptives and zero-order correlations with related concepts.

	FI-facts	Need for evidence	Truth is political	n
**Study 2 (2016 OSoC)**				
*Descriptives:*				
M (SD)	5.52 (1.56)	5.88 (1.24)	4.55 (1.89)	606
Min.—Max.	1–9	1.8–9	1–9	606
Skew	-.24	.-46	.08	606
Kurtosis	.13	.11	-.41	606
*Zero-order correlations:*				
Ideology (Conservatism)	.16**	-.04	.22**	606
Party (Republican affiliation)	.08*	-.05	.19**	606
Trump supporter	.11**	.07	.20**	606
Education	-.13**	.18**	-.11**	606
Political interest	-.11**	.23**	-.04	606
Religious fundamentalism^a^	.22**	-.24**	.23**	507
**Study 3 (2016 NSF, wave 1)**				
*Descriptives:*				
M (SD)	3.28 (.64)	3.74 (.71)	3.03 (.77)	947
Min.—Max.	1–5	1–5	1–5	947
Skew	.02	-.34	-.04	947
Kurtosis	.61	.23	.15	947
*Zero-order correlations:*				
Ideology (Conservatism)	.12**	-.09**	.17**	942
Party (Republican affiliation)	.08*	-.04	.19**	942
Trump supporter	.10*	-.00	.25**	947
Conservative site use	.04	.06	.08*	947
Liberal site use	-.03	.15**	-.08*	947
Political interest	-.05	.23**	.01	944
Education	-.05	.21**	-.08*	947
Need for Cognition	-.20**	.11**	-.21**	947

^a.^ Trait measured in a subsequent wave.

* *p* < .05,

** *p* < .01

### Conspiracist ideation

#### Visual inspection

**Fig 1:**
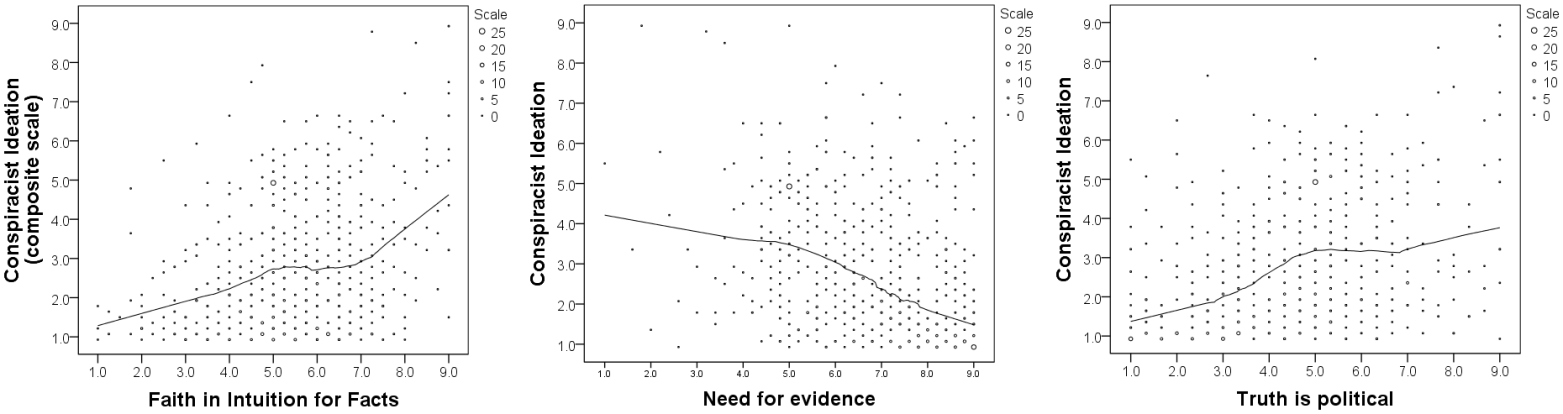
Scatterplots with locally weighted regression lines. Values shown are for composite scales. Size of marker corresponds to number of cases. Fit lines drawn using iterative least squares (Loess) with 50% of the data points to calculate the local smoother via the Epanechnikov kernel function. Fit lines suggest a modest linear relationship between conspiracist ideation and each of the three epistemic beliefs.

We begin our examination of the potential relationship between epistemic beliefs and conspiracist ideation with a visual inspection of corresponding scatterplots. The plots are overlaid with locally weighted regression lines to help illustrate trends in the data (see Fig 1). The relationships appear linear, and in the anticipated direction: *Need for evidence* appears negatively correlated with conspiracist ideation, while *FI-facts* and *Truth is political* both appear positively correlated.

#### Latent regression analysis

We use structural equation modeling (SEM) to examine epistemic beliefs’ contribution to conspiracist ideation more rigorously. The model, which uses the 2016 OSoC data, is based on latent variables wherever possible, which allows it to better account for measurement error, thereby yielding more precise estimates of regression model parameters.

**Fig 2:**
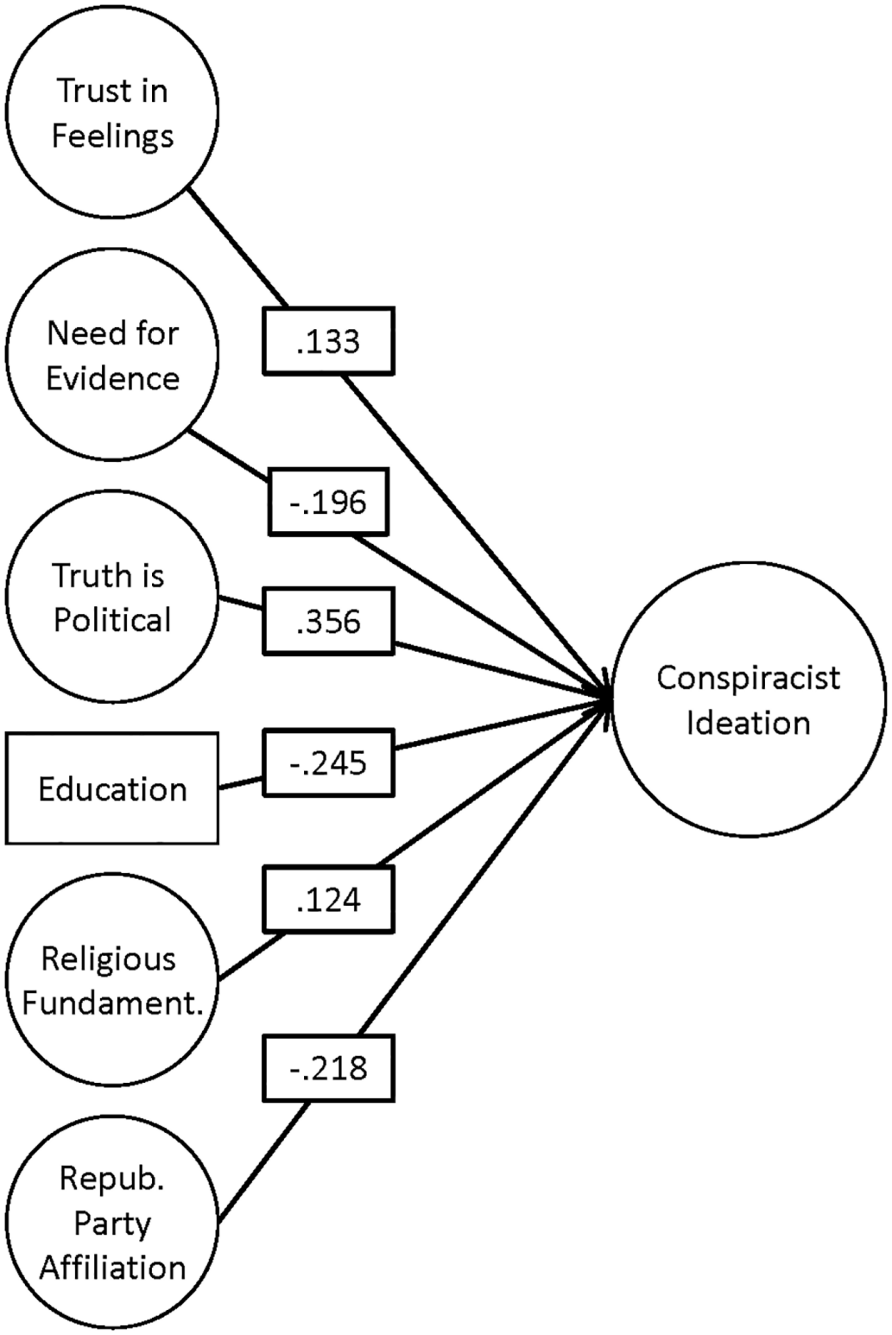
Structural equation model summarizing factors associated with conspiracist ideation. Circles denote latent variables; education is manifest. All links shown are standardized and significant; *p* < .02. Except for education, manifest variables and their loadings are not shown. Disturbances on endogenous factors are also omitted from the figure. Path coefficients for all three epistemic beliefs measures indicate that these factors have an influence on conspiracist ideation that is comparable to or larger than other established predictors.

In order to demonstrate the unique explanatory power of epistemic beliefs, we include several predictors shown in other work to influence conspiracist ideation, including religious fundamentalism [17], political identity [11], and education (see Fig 2). Education is the only manifest variable in the model, and all predictors were allowed to correlate. Epistemic beliefs and conspiracist ideation were measured in the same wave, and we impute missing data using full information maximum-likelihood methods via Mplus [48]. Model fit is good, *CFI* = .96, *RMSEA* = .04, *SRMR* = .05, and adding epistemic beliefs to the model yields a significant improvement in fit, change in *χ*^2^ = 390.11, *df* = 203, *p* < .001. The influence of each of the three factors is modest but significant. As expected, path coefficients indicate that *FI-facts* and *Truth is political* promote conspiracist ideation, while *Need for evidence* constrains it. The variance of conspiracist ideation explained is *R*^2^ = 0.42 after including the (latent) epistemological beliefs measures, up from *R*^2^ = 0.22 without them.

## Beliefs about high-profile issues

Americans frequently hold inaccurate beliefs about scientific and political facts, regardless of their belief in conspiracy theories. We next examine whether epistemic beliefs help explain this alternative form of misperception. We consider the accuracy of respondents’ beliefs about four issues commonly associated with misperception in the U.S.: anthropogenic climate change, Muslims’ opposition toward violence directed at the West, the absence of WMDs in Iraq prior to the U.S.-led invasion, and the safety of vaccination.

Scatterplots for these issues are generally comparable to those reported for conspiracist ideation (see S3, S4 and S5 Figs), though visual inspection suggests that the relationship between epistemic beliefs and issue belief accuracy may be weaker than between beliefs and conspiracist ideation. The scatterplots also suggest that the nature of relationships may vary modestly between issues (at least one appears as though it might be curvilinear).

We again use SEM-based latent regression analysis to test the statistical significance of these relationships. Consistent with our causal argument, we model issue beliefs measured in the third wave as a product of epistemic beliefs measured in the second. Our expectations are substantively unchanged from the model of conspiracist ideation. All four models achieve good fit, and, as with the model of conspiracist ideation, epistemic beliefs yield a significant improvement in fit, though the magnitude of improvement is smaller (Table 6). After including all predictors, the models account for between 25% and 30% of the variance in respondents’ beliefs about climate change, Muslims’ attitudes toward violence, and WMDs; however, the model of vaccine safety only explains about 15% of the variance. All four models offer a significant improvement in model fit over those based on a host of other contributors, including the well documented influence of media exposure [33, 49]. *Need for evidence* and *Truth is political* operate as expected, but *FI-facts* was only significant when modeling beliefs about WMDs, suggesting that this factor has a more consistent influence on beliefs that are explicitly related to conspiracist ideation.

**Table 6:** Structural equation models summarizing factors associated with lagged issue accuracy.

	Climate change	Muslim attitudes	WMDs in Iraq	Vaccine safety
*FI-facts*	0.07 (.05)	-0.02 (.05)	-0.10 (.05)*	-0.05 (.05)
*Need for evidence*	0.16 (.04)***	0.20 (.05)***	0.19 (.05)***	0.21 (.05)***
*Truth is political*	-0.12 (.04)*	-0.20 (.05)***	-0.07 (.05)	-0.17 (.05)**
Ideology (Conservatism)	-0.36 (.04)***	-0.30 (.04)***	-0.28 (.04)***	-0.06 (.04)
Political attention	-0.07 (.05)	0.07 (.05)	0.08 (.05)	0.15 (.05)**
# cons. sites used^a^	-0.21 (.04)***	-0.07 (.04)	-0.14 (.04)**	-0.07 (.04)
# lib. sites used^a^	0.14 (.03)**	0.02 (.04)	0.15 (.04)***	0.02 (.05)
Education^a^	0.02 (.04)	0.05 (.04)	0.00 (.04)	0.03 (.04)
Need for cognition	-0.04 (.05)	-0.03 (.06)	-0.02 (.06)	-0.05 (.05)
Goodness of Fit	*CFI* = .96	*CFI* = .96	*CFI* = .97	*CFI* = .96
	*RMSEA* = .04	*RMSEA* = .04	*RMSEA* = .04	*RMSEA* = .04
	*SRMR* = .04	*SRMR* = .04	*SRMR* = .04	*SRMR* = .04
GoF improvement:	*χ*^2^ = 323.43,	*χ*^2^ = 307.42,	*χ*^2^ = 297.11,	*χ*^2^ = 207.20,
Epistemic beliefs	*df* = 174, *p* < .001	*df* = 174, *p* < .001	*df* = 174, *p* < .001	*df* = 174, *p* < .01
*R*^2^	0.31	0.26	0.25	0.15
Δ*R*^2^: Epistemic beliefs	0.03	0.06	0.04	0.06
*n*	625	625	625	625

Outcomes are measured in wave 3, epistemic beliefs in wave 2. Cell values show standardized coefficients (SE).

^a.^ Manifest variables (all others are latent).

* *p* < .05,

** *p* < .01,

*** *p* < .001

We also test models that includes a quadratic term associated with *Need for evidence* when estimating WMD beliefs based on the curvilinear relationship suggested by the corresponding scatterplot (see lower-left panel of S4 Fig). The quadratic term is significant (see S2 Table), but the improvement in model fit is small, *AIC*_linear_ = 40842.73 versus *AIC*_polynomial_ = 40841.04, and the only notable change in model coefficients is that *FI-facts* is no longer significant, *β* = −.18, *p* = .085. Having no theoretical reason to expect the non-linear relationship, we view this result cautiously.

### Conditioning on ideology

To examine whether the influence of the new measures are conditioned on political respondent ideology, we re-estimate the models for all four outcomes, adding interaction terms between ideology and each of the three epistemic beliefs. Of the 12 interaction terms, only two are significant (see S3 Table for all model coefficients). When predicting accuracy about WMDs, *Need for evidence* is less positively associated with accuracy the more conservative an individual is, *β* = −.10, *p* = .012. In the model of beliefs about Muslims, *Truth is political* is more strongly associated with inaccuracy as conservativism increases, *β* = −.12, *p* = .005. These results are consistent with the idea that epistemic beliefs can be deployed in defense of an ideological position, but most of the interactions are not significant. No effects are conditioned on ideology for either climate change or vaccine safety, and even after accounting for the significant interaction term, *Truth is political* is associated with a decrease in accuracy about Muslims among even the most liberal respondents.

## Discussion

Scientific and political misperceptions are dangerously common in the U.S. today. The willingness of large minorities of Americans to embrace falsehoods and conspiracy theories poses a significant threat to society’s ability to make well informed decisions about pressing challenges. We develop and validate three scales that measure differences in individuals’ views about the nature of knowledge and knowing. Accounting for these epistemic beliefs substantively improves our ability to estimate the accuracy of individuals’ beliefs. We find that individuals who trust their intuition, putting more faith in their ability to use intuition to assess factual claims than in their conscious reasoning skills, are uniquely likely to exhibit conspiracist ideation. Those who maintain that beliefs must be in accord with available evidence, in contrast, are less likely to embrace conspiracy theories, and they are less likely to endorse other falsehoods, even on politically charged topics. Finally, those who view facts as inexorably shaped by politics and power are more prone to misperception than those who believe that truth transcends social context. These individual-difference measures are fairly stable over time. Although the influence of epistemic beliefs is sometimes conditioned on ideology, this is the exception; in most instances the two types of factors operate independent of one another.

### Theoretical implications

Epistemic beliefs are an understudied tool for understanding misperceptions, and the short scales developed here augment the rich and evolving toolkit available to scholars working in this area. A complex set of mechanisms lead individuals to endorse falsehoods, including political motivations [7], flawed (meta)cognitive strategies [14, 15], social dynamics [12], psychological predispositions [17], and media effects [13]. The present research demonstrates that individuals’ beliefs about the nature of knowledge also play an important role. Although the complexity of this theoretical story is daunting, accounting for these myriad processes appears essential to making sense of persistent misperceptions.

We see epistemic beliefs as complementing other measures of thinking-style that are associated with belief (in)accuracy, notably including cognitive reflection and numeracy [12]. The existing measures emphasize ability: individuals who are capable of thinking carefully through problems, and who work with numbers fluently are more likely to find ways to reconcile new information with their existing beliefs. Epistemic beliefs, in contrast, are focused more on how the individual thinks about the nature of knowledge and the process of knowing. These traits are not concerned with ability, and their contribution to our understanding of misperceptions is distinct.

There is some evidence that the various mechanisms for explaining misperceptions interact in important ways. We find modest evidence that individuals may at times deploy their epistemic beliefs strategically, meaning that epistemological beliefs are more influential when they align with the individual’s political predispositions. In most cases, however, we find that the influence of epistemic beliefs do not vary by ideology, and even in instances when ideology and epistemic beliefs interact, the direction of epistemic beliefs’ influence remains the same even as the magnitude varies. These factors are not merely conduits for expressing political predispositions.

Finally, we do not believe that these findings contradict recent work in neuroscience and social psychology suggesting that non-conscious bodily experiences are useful and necessary to humans’ ability to form judgments [14, 24]. The present research does, however, suggest that reliance on feelings entails considerable risk. Individuals’ willingness to weigh evidence and to test the logic supporting a claim is an important check against biased instincts and flawed intuition.

### Practical implications

Cumulatively, these results suggest that efforts aimed at shifting citizens’ epistemic beliefs might be a useful complement to other, more direct accuracy improvement strategies. Individuals’ beliefs do not uniformly align with the knowledge they hold about relevant evidence, and epistemic beliefs may help to explain how such inconsistencies can persist. If educators, science communicators, fact checkers, and journalists are able to convince individuals to place more weight on reason and evidence, and less on intuition and instinct, and if individuals can be persuaded that empirical reality provides a strong check against political manipulation, then it is plausible that citizens might become more responsive to accurate information about the political and scientific world. These strategies complement previously identified tactics designed to account for worldview differences [8] or social pressures [27], and they resemble other approaches based on thinking style, such as encouraging analytic thinking to reduce conspiracist ideation [32].

### Limitations and future research

This study provides evidence that epistemic beliefs can influence the accuracy of individuals’ perceptions, but there are important limitations and several open questions. First, these results are based on self-reported data, which is prone to bias. Individuals may lack insight into their own behaviors [50] and in some cases may be inclined to misrepresent their beliefs [51]. Given this, we assume that respondents’ endorsement of falsehoods in these surveys represents a complex amalgam of belief, identity expression, and political strategy. Nevertheless, self-reports remain an important means of assessing beliefs. What people say they believe matters. Understanding why people endorse the beliefs they do is an important part of the larger misperception puzzle. It would, however, be informative to compare these results to studies that rely on behavioral measures corresponding to epistemic beliefs and/or to issue beliefs revealed through unobtrusive observation.

Another limitation stems from the fact that factual beliefs were consistently assessed prior to measuring epistemic beliefs. It is possible that this ordering might artificially inflate the correlation between epistemic and factual beliefs if people are trying to rationalize the claims they just endorsed. We believe this is unlikely: respondents were given no indication that their beliefs were inaccurate, so there was little incentive for rationalization. Further, Study 3 presented several questions between the measures of epistemic and factual beliefs, reducing the chance that the items influenced one another. Nonetheless, replicating these results after reversing the order of the items would provide a useful confirmation.

Another important next step would be to replicate these results using other measures of conspiracist ideation, and to test epistemic beliefs contribution in the context of other recently documented predictors [17, 20]. Placing these alternative approaches in conversation with one another would provide important insight into the relative strength of different explanations.

Given that the primary objective of this work was to develop and test a new set of scales, there is much about the influence of epistemic beliefs that remains unexplored. Our heavy reliance on cross-sectional data greatly limits our ability to draw causal conclusions. Although we have reason to think that the attributes identified here will moderate individuals’ response to factually accurate information, experimental work and panel data are needed to test this assertion. The fact that epistemic beliefs exhibit modest change over time also raises important questions. Political stakeholders have systematically promoted the idea that political biases are endemic to science and journalism [52, 53], which could result in the entanglement of epistemic beliefs and political identity. These issues merit additional research.

## Conclusions

Epistemic beliefs represent an important theoretical approach to understanding misperceptions. They complement existing explanations, and contribute to our ability to explain why individuals endorse falsehoods. The short scales offered here provide a straightforward way of accounting for these individual differences when modeling misperceptions. The influence that these factors have on political and scientific misperceptions and, especially, on conspiracist ideation suggest a variety of novel real-world strategies for countering such inaccuracies.

## Supporting information

S1 Appendix Sample descriptives. (PDF)

S2 Appendix Measurement item generation, question wording, and descriptives. (PDF)

S1 Table EFA factor loadings (2015). (PDF)

S2 Table Structural equation models summarizing factors associated with lagged WMD accuracy, including quadratic term. (PDF)

S3 Table Structural equation models summarizing factors associated with lagged issue accuracy, including interactions with political ideology. (PDF)

S1 Fig Typical CFA factor loadings for epistemic belief items. (PDF)

S2 Fig Frequency distributions of composite scores for three epistemic beliefs. (PDF)

S3 Fig Scatterplots of *FI-facts* by accuracy with locally weighted regression lines. (PDF)

S4 Fig Scatterplots of need for evidence by accuracy with locally weighted regression lines. (PDF)

S5 Fig Scatterplots of truth is political by accuracy with locally weighted regression lines. (PDF)
