# Supplementary material for: Epistemic beliefs’ role in promoting misperceptions and conspiracist ideation
Source: PLoS One. 2017 Sep 18;12(9):e0184733. doi: 10.1371/journal.pone.0184733 (PMC5603156; doi:10.1371/journal.pone.0184733)
Supplement: S1 Appendix — (PDF) [file pone.0184733.s001.pdf]

S1 Appendix. Sample descriptives.

2015 Omnibus Survey for the School of Communication (OSoC)

We use weights, computed by GfK, the company that administered the survey, to more closely approximate a representative sample when reporting survey demographics and estimating population parameters throughout. Race-ethnicity composition is typical of the US: 65.5% White, Non-Hispanic; 15.2% Hispanic; 11.5% Black, Non-Hispanic; with smaller numbers of respondents identifying with other or multiple categories. Educational attainment is also comparable to national averages: 12.4% less than high school, 58.4% high school or some college, and 29.2% Bachelor's degree or higher. The sample leans Democratic (44.9% Republican versus 50.9% Democratic), but liberals are in the minority (35.4% conservative, 36.4% moderate, and 25.7% liberal). The median household income was between $60,000 and $74,999, which is somewhat higher than the national median (about $54K according to the 2014 American Community Survey). The survey completion rate was 60%.

2016 OSoC

This was a three-wave survey, with epistemic beliefs and conspiracist ideation measured in wave 2. In that wave, 64.4% of the sample was White, Non-Hispanic, 11.8% was Black, Non-Hispanic, 15.7% was Hispanic, and the remainder identified with other racial and ethnic categories. Education levels reflect national averages with 11.7% less than high school, 29.0% high school, 28.6% some college, and 30.8% Bachelor's degree or higher. The sample includes 37.4% identifying as Republicans, 51.9% as Democrats, and 10.7% Independents. Ideologically, the sample is 41.0% conservative, 24.9% moderate, and 34.0% liberal. The median annual household income was between $60,000 and $74,999. The completion rate for the first wave of the survey was 52.5%

2016 National Science Foundation funded (NSF) survey

This was also a three-wave survey, and epistemic beliefs and science/political beliefs were measured in all waves. In the first wave 64.9% of the sample was White, Non-Hispanic, 11.7% was Black, Non-Hispanic, 15.5% were Hispanic, with the remaining 7.9% identifying with a different race or ethnicity. Education levels were similar to the two other surveys, with 12.3% less than high school, 29.6% high school, 28.3% some college, and 29.8 Bachelor's degree or higher. The sample includes 36.1% identifying as Republicans, 42.5% as Democrats, and 21.5% Independents. Ideologically, the sample is 35.1% conservatives, 35.0% moderate, and 29.9% liberals. The mean annual household income was between $60,000 and $74,999. The completion rate for the first wave was 62%.
