# Supplementary material for: Epistemic beliefs’ role in promoting misperceptions and conspiracist ideation
Source: PLoS One. 2017 Sep 18;12(9):e0184733. doi: 10.1371/journal.pone.0184733 (PMC5603156; doi:10.1371/journal.pone.0184733)
Supplement: S2 Appendix — (PDF) [file pone.0184733.s002.pdf]

S2 Appendix. Measurement item generation, question wording, and descriptives.

Epistemic belief candidate-item generation

To develop the initial set of candidate items for these scales, we first looked both to prior theoretical work and to existing scales used to assess related concepts, notably the Myers-Briggs Personality Type Indicator (MBTI) (1), and the Faith in Intuition (FI) scale (2). Although MBTI and FI differ in their emphasis on the source and target of "intuition" and "feeling," the conceptual work and associated scale items have clear relevance to both the *FI-feelings* and *Need for evidence* scales. Several variants on the original FI scale were included in our candidate items. To draft the *Truth is political* items, which focus on the social construction of reality, the existence of objective truths, and the nature of facts, we turned to theoretical work by Baym (3) that identifies numerous postmodernist attitudes and beliefs that align well with this concept.

After developing an initial set of items for all three concepts, we recruited four scholars who were otherwise uninvolved in the research to help refine the list of candidate items. Items judged to be highly relevant by all evaluators were retained; those receiving more mixed support were revised; and the least relevant items were dropped. Once we had a list of items that were conceptually focused on the topics of interest, we iteratively refined item wording through a series of tests using a convenience sample of participants recruited via opt-in online panels (Amazon's MTurk and Qualtrics Panels). Items exhibiting very low correlation with others in the same scale were dropped, and wording for other items was revised. The scales item ultimately selected are one-sided because the reverse-coded items did not load as strongly in the factor analyses.

Epistemic belief question wording and descriptives

2015 Omnibus Survey for the School of Communication (OSoC).

Twenty potential measurement items were presented on a 1 (strongly disagree) to 7 (strongly agree) scale:

1. Facts depend on their political context
2. What is true is influenced by the social setting
3. Facts are dictated by those in power
4. here are multiple truths in every situation
5. The truth is relative
6. What counts as truth is defined by power
7. Scientific conclusions are shaped by politics
8. Scientific results are free of political influence
9. The political situation doesn't change the facts
10. The truth is absolute and knowable
11. I believe what I want to believe, no matter what the evidence is
12. I trust my gut to tell what's true and what's not

13. I am a very intuitive person
14. I can usually feel when a claim is true or false even if I can't explain how I know
15. I trust my initial feelings about the facts
16. My initial impressions are almost always right
17. Evidence is more important than whether something feels true
18. I trust the facts, not my instincts, to tell me what is true
19. A hunch needs to be confirmed with data
20. I need to be able to justify my beliefs with evidence

2016 OSoC

Epistemic beliefs were assessed using 11 items that emerged from the EFA using the 2015 OSoC data. Respondents were asked how much they personally agreed with the series of items using a 1 (*Strongly Disagree*) to 9 (*Strongly Agree*) scale. Four items were used to measure *FI-facts* (*alpha* = .797; *mean* = 5.47; *SD* = 1.56) and *need for evidence* (*alpha* = .825; *mean* = 5.95; *SD* = 1.20), while a three item scale measured *truth is political* (*alpha* = .768; *mean* = 4.50; *SD* = 1.94). The fourth item assessing truth is political ("Facts depend on their political context") was unintentionally omitted from the battery by the company administering the survey. Although we are reporting statistics describing composite scores throughout this document, all epistemic belief items were entered as manifest indicators of latent concepts in the SEM.

2016 National Science Foundation funded (NSF)

Epistemic beliefs were assessed in Waves 1-3 using the 12 items selected based on the EFA using the 2015 OSoC data. Respondents were asked how much they agreed with the series of items using a 1 (strongly disagree) to 5 (strongly agree) scale. Four items were used to measure *FI-facts* (Wave 1: *alpha* = .780; *mean* = 3.25; *SD* = .65; Wave 2: *alpha* = .791; *mean* = 3.22; *SD* = .64; Wave 3: *alpha* = .791; *mean* = 3.26; *SD* = .65), *need for evidence* (Wave 1: *alpha* = .812; *mean* = 3.73; *SD* = .72; Wave 2: *alpha* = .826; *mean* = 3.71; *SD* = .74; Wave 3: *alpha* = .829; *mean* = 3.80; *SD* = .71), and *truth is political* (Wave 1: *alpha* = .747; *mean* = 3.02; *SD* = .78; Wave 2: *alpha* = .806; *mean* = 3.00; *SD* = .81; Wave 3: *alpha* = .872; *mean* = 2.92; *SD* = .79) in each Wave.

Conspiracist ideation question wording and descriptives

In the 2016 OSoC survey respondents' were asked to report their belief in nine prominent conspiracy theories measured using a 1 (definitely not true) to 9 (definitely true). Two items were dropped to improve the reliability of the scale ("The alleged link between second-hand tobacco smoke and ill health is based on bogus science and is an attempt by a corrupt cartel of medical researchers to replace rational science with dogma") and to avoid overlap with subsequent analyses ("The claim that the climate is changing due to emissions from fossil fuels is a hoax perpetrated by corrupt scientists who wish to spend more taxpayer money on climate research"). The resulting seven items (see Table 2 for wording) were used to create the (latent) conspiracist ideation variable (*alpha* = .807; *mean* = 2.76; *SD* = 1.65).

Issue belief question wording and descriptives

The 2016 NSF survey measured belief accuracy about four prominent claims related to political and scientific issues. Respondents were presented pairs of contrasting statements and asked to place a mark on a five-point scale closer to the statement that best described their personal beliefs, placing the mark in the middle if they were unsure of the truth. Responses were recoded following data collection so that higher scores represent greater accuracy, resulting in a scale ranging from 1 (most inaccurate) to 5 (most accurate). Statements were presented as follows: ("Human activity is contributing to changes in the global climate—Human activity has no influence on global climate," *mean* = 3.69; *SD* = 1.23; "Most Muslims support violence against Western countries, including the U.S.—Most Muslims oppose violence against Western countries, including the U.S.," *mean* = 3.46; *SD* = 1.23; "Iraq had weapons of mass destruction immediately before the Iraq war began—Iraq had no weapons of mass destruction immediately before the Iraq war began," *mean* = 3.13; *SD* = 1.34; "Vaccines cause autism—Vaccination is unrelated to autism," *mean* = 3.75; *SD* = 1.19.

Control variable question wording and descriptives

2016 OSoC

A series of variables known to be associated with conspiracist ideation were included as controls. Education was measured on a 14-point scale, from "No formal education" (1) to "Professional or Doctorate degree" (14), with a *median* score of 10 ("Some college, no degree"). Also included was religious fundamentalism, which was measured in the third wave by asking participants how much they agreed with a series of statements on a scale from -4 (strongly disagree) to 4 (strongly agree). Statements included ("God has given humanity a complete, unfailing guide to happiness and salvation, which must be totally followed"; "The basic cause of evil in this world is Satan, who is still constantly and ferociously fighting against God"; "There is a particular set of religious teachings in this world that are so true, you can't go any 'deeper' because they are the basic, bedrock message that God has given humanity"; "When you get right down to it, there are basically only two kinds of people in the world: the Righteous, who will be rewarded by God; and the rest, who will not"; "To lead the best, most meaningful life, one must belong to the one, fundamentally true religion"; "The fundamentals of God's religion should never be tampered with, or compromised with others' true beliefs" (*alpha* = .917; *mean* = -.530; *SD* = 2.49). Political interest was measured on a four-point scale from "Not at all" (1) to "Very" (4) (*mean* = 2.71; *SD* = 1.03). Ideology was measured on a 9-point scale, from "Very liberal" (1) to "Very conservative" (*mean* = 5.31; *SD* = 2.27). 38.2% of respondents identified as Trump supporters. Finally, political party affiliation was measured on a scale from 1 (*Strong Democrat*) to 7 (*Strong Republican*) (*mean* = 4.81; *SD* = 2.88).

2016 NSF

The survey included several variables that are often associated with issue belief accuracy. Education was measured on a 14-point scale, from "No formal education" (1) to "Professional or Doctorate degree" (14), with a *median* score of 10 ("Some college, no degree"). Political ideology was measured with questions measuring general political views, as well as views on

economic and social issues, on a scale from 1 (Very Liberal) to 7 (Very Conservative) (*alpha* = .941; *mean* = 4.14; *SD* = 1.52). 39.5% of respondents identified as Trump supporters. Political interest was captured by two items ("In general, how interested are you in politics and public affairs?"; "How closely do you follow news about the 2016 Presidential election?") measured on a 1 ("Not at all") to 4 ("Very") scale (*alpha* = .742; *mean* = 2.72; *SD* = .90). Partisan websites use was measured by providing two lists of twenty prominent conservative and liberal websites or mobile apps and asking respondents if they'd used the site at least once in the last month. We then summed the number of liberal (*mean* = .86; *SD* = 1.61) and conservative (*mean* = .78; *SD* = 1.61) sites used. Finally, *Need for Cognition* was measured using 3 items ("I dislike questions which could be answered in many different ways"; "I feel irritated when one person disagrees with what everyone else in a group believes"; "I do not usually consult many different opinions before forming my own view") on a 1 (*Strongly Disagree*) to 6 (*Strongly Agree*) scale (*alpha* = .56; *mean* = 3.83; *SD* = .85).

References

1. Quenk NL. Myers-Briggs Type Indicator Assessment. 2nd ed. Hoboken, NJ, USA: Wiley; 2009.
2. Epstein S, Pacini R, Denes-Raj V, Heier H. Individual differences in intuitive–experiential and analytical–rational thinking styles. Journal of Personality and Social Psychology. 1996;71(2):390-405.
3. Baym G. From Cronkite to Colbert: the evolution of broadcast news. Boulder: Paradigm Publishers; 2010.
