# Supplementary material for: Epistemic beliefs’ role in promoting misperceptions and conspiracist ideation
Source: PLoS One. 2017 Sep 18;12(9):e0184733. doi: 10.1371/journal.pone.0184733 (PMC5603156; doi:10.1371/journal.pone.0184733)
Supplement: S1 Table — (PDF) [file pone.0184733.s003.pdf]

S1 Table. EFA Factor Loadings (2015)

GEOMIN ROTATED LOADINGS (* significant at 5% level)

	1	2	3
FEEL1	**0.75***	-0.10	0.02
FEEL2	**0.61***	-0.08	0.00
FEEL3	**0.73***	0.02	-0.02
FEEL4	**0.69***	0.15*	-0.02
EVID1	-0.01	**0.82***	0.03
EVID2	-0.21*	**0.68***	-0.03
EVID3	0.08	**0.71***	0.03
EVID4	0.14*	**0.60***	0.01
POLI1	0.01	-0.07	**0.60***
POLI2	-0.01	0.03	**0.72***
POLI3	0.01	0.06	**0.72***
POLI4	-0.07	-0.03	**0.61***
