# Supplementary material for: Epistemic beliefs’ role in promoting misperceptions and conspiracist ideation
Source: PLoS One. 2017 Sep 18;12(9):e0184733. doi: 10.1371/journal.pone.0184733 (PMC5603156; doi:10.1371/journal.pone.0184733)
Supplement: S2 Table — (PDF) [file pone.0184733.s004.pdf]

S2 Table. Structural Equation Models summarizing factors associated with lagged WMD accuracy, including quadratic term

	**WMDs in Iraq**	**WMDs in Iraq**
FI-facts	-0.10 (.05)*	-0.18 (.10)
Need for evidence	0.19 (.05)***	0.42 (.10)***
Need for evidence, squared	—	0.15 (.06) *
Truth is political	-0.07 (.05)	-0.09 (.08)
Ideology (Conservatism)	-0.28 (.04)***	-0.26 (.04)***
Political attention	0.08 (.05)	0.13 (.09)
# cons. sites used [a]	-0.14 (.04)**	-0.11 (.03)***
# lib. sites used [a]	0.15 (.04)***	0.12 (.03)***
Education [a]	0.00 (.04)	0.00 (.03)
Need for cognition	-0.02 (.06)	-0.03 (.11)
AIC	40842.73	40841.04
BIC	41344.19	41346.95
n	625	625

Outcomes are measured in wave 3, epistemic beliefs in wave 2.
Cell values show standardized coefficients (SE). a. Manifest variables (all others are latent).
* $p < .05$, ** $p < .01$, *** $p < .001$
