# Supplementary material for: Epistemic beliefs’ role in promoting misperceptions and conspiracist ideation
Source: PLoS One. 2017 Sep 18;12(9):e0184733. doi: 10.1371/journal.pone.0184733 (PMC5603156; doi:10.1371/journal.pone.0184733)
Supplement: S3 Table — (PDF) [file pone.0184733.s005.pdf]

S3 Table. Structural Equation Models summarizing factors associated with lagged issue accuracy, including interactions with political ideology

	**Climate change**	**Muslim attitudes**	**WMDs in Iraq**	**Vaccine safety**
FI-facts (FIF)	0.07 (.05)	-0.02 (.05)	-0.10 (.05)*	-0.05 (.05)
**FIF X Ideology**	—	—	—	—
Need for evidence (NFE)	0.16 (.04)***	0.21 (.05)***	0.18 (.05)***	0.21 (.05)***
**NFE X Ideology**	—	—	**-0.10 (.04)***	—
Truth is political (TIP)	-0.12 (.04)*	-0.21 (.05)***	-0.06 (.05)	-0.17 (.05)**
**TIP X Ideology**	—	**-0.12 (.04)****	—	—
Ideology (Conservatism)	-0.36 (.04)***	-0.30 (.04)***	-0.27 (.04)***	-0.06 (.04)
Political attention	-0.07 (.05)	0.09 (.05)	0.08 (.05)	0.15 (.05)**
# cons. sites used [a]	-0.21 (.04)***	-0.06 (.05)	-0.13 (.04)***	-0.07 (.04)
# lib. sites used [a]	0.14 (.03)**	0.01 (.04)	0.14 (.04)***	0.02 (.05)
Education [a]	0.02 (.04)	0.05 (.04)	0.00 (.04)	0.03 (.04)
Need for cognition	-0.04 (.05)	-0.03 (.06)	-0.02 (.06)	-0.05 (.05)
R^2	.31	.29	.24	.15
ΔR^2: Interaction	.00	.03	-.01	.00
n	625	625	625	625

Outcomes are measured in wave 3, epistemic beliefs in wave 2.
Cell values show standardized coefficients (SE). Significant interaction coefficients in bold; non-significant interactions terms omitted from models.
[a]Manifest variables (all others are latent).
* $p < .05$, ** $p < .01$, *** $p < .001$
