# Supplementary material for: Epistemic beliefs’ role in promoting misperceptions and conspiracist ideation
Source: PLoS One. 2017 Sep 18;12(9):e0184733. doi: 10.1371/journal.pone.0184733 (PMC5603156; doi:10.1371/journal.pone.0184733)

S1 Fig. Typical CFA Factor Loadings for epistemic belief items

All links shown are standardized and significant; $p < .001$. Results from NSF 2016, Wave 1 shown.

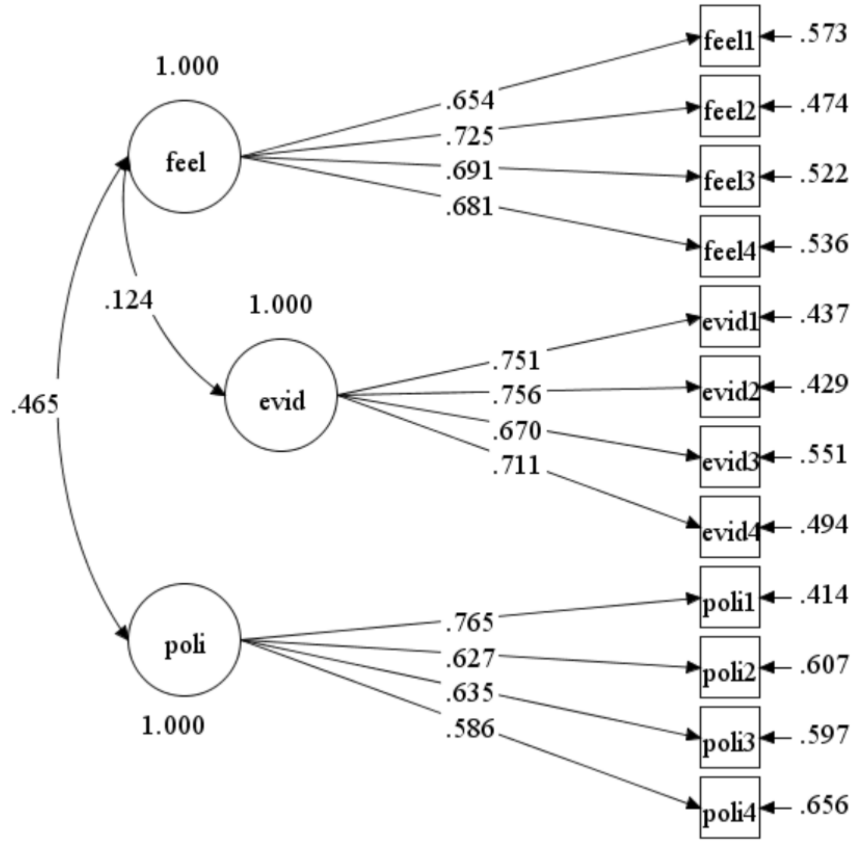

Supplement: S1 Fig — (PDF) [file pone.0184733.s006.pdf]
