# Supplementary material for: Epistemic beliefs’ role in promoting misperceptions and conspiracist ideation
Source: PLoS One. 2017 Sep 18;12(9):e0184733. doi: 10.1371/journal.pone.0184733 (PMC5603156; doi:10.1371/journal.pone.0184733)

S2 Fig. Frequency distributions of composite scores for three epistemic beliefs

Each histogram shows the distribution of composite scale scores. Each variable represents the average response across the four constituent items on a five-point scale from 'Strongly agree' (1) to 'Strongly disagree' (5). Results from NSF 2016, wave 1 shown.

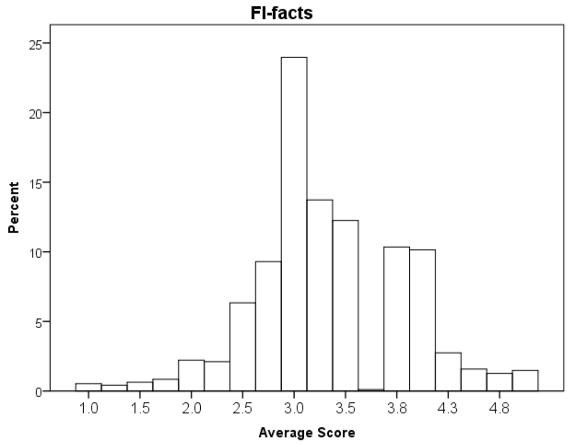

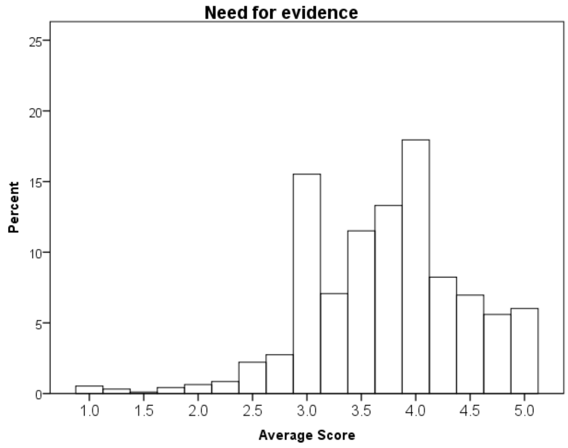

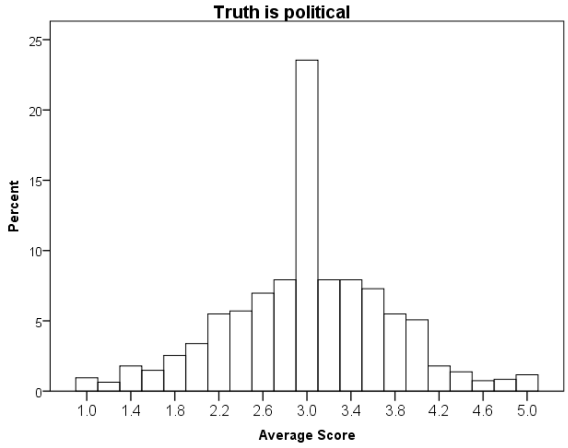

Supplement: S2 Fig — (PDF) [file pone.0184733.s007.pdf]
