# Supplementary material for: Epistemic beliefs’ role in promoting misperceptions and conspiracist ideation
Source: PLoS One. 2017 Sep 18;12(9):e0184733. doi: 10.1371/journal.pone.0184733 (PMC5603156; doi:10.1371/journal.pone.0184733)

S3 Fig. Scatterplots of FI-facts by accuracy with locally weighted regression lines

Values shown are for composite scales. Size of marker corresponds to number of cases. Fit line drawn using iterative least squares (Loess) with 50% of the data points to calculate the local smoother via the Epanechnikov kernel function. There is little evidence that *FI-facts* influences issue accuracy.

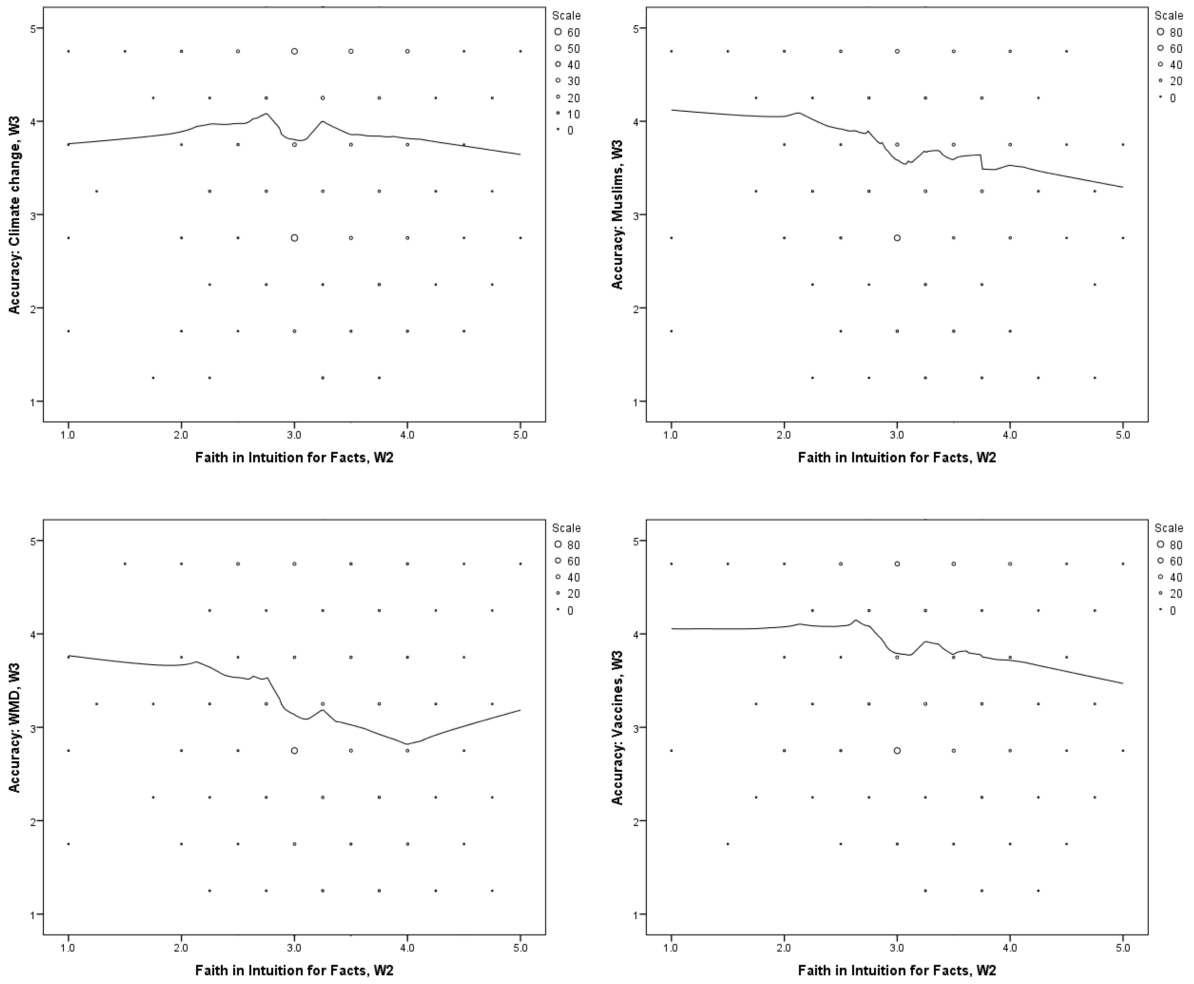

Supplement: S3 Fig — (PDF) [file pone.0184733.s008.pdf]
