# Supplementary material for: Epistemic beliefs’ role in promoting misperceptions and conspiracist ideation
Source: PLoS One. 2017 Sep 18;12(9):e0184733. doi: 10.1371/journal.pone.0184733 (PMC5603156; doi:10.1371/journal.pone.0184733)

S4 Fig. Scatterplots of Need for Evidence by accuracy with locally weighted regression lines

Values shown are for composite scales. Size of marker corresponds to number of cases. Fit line drawn using iterative least squares (Loess) with 50% of the data points to calculate the local smoother via the Epanechnikov kernel function. Fit lines suggest a relationship between *Need for evidence* and issue accuracy, though it may in some cases be non-linear (e.g., WMD beliefs).

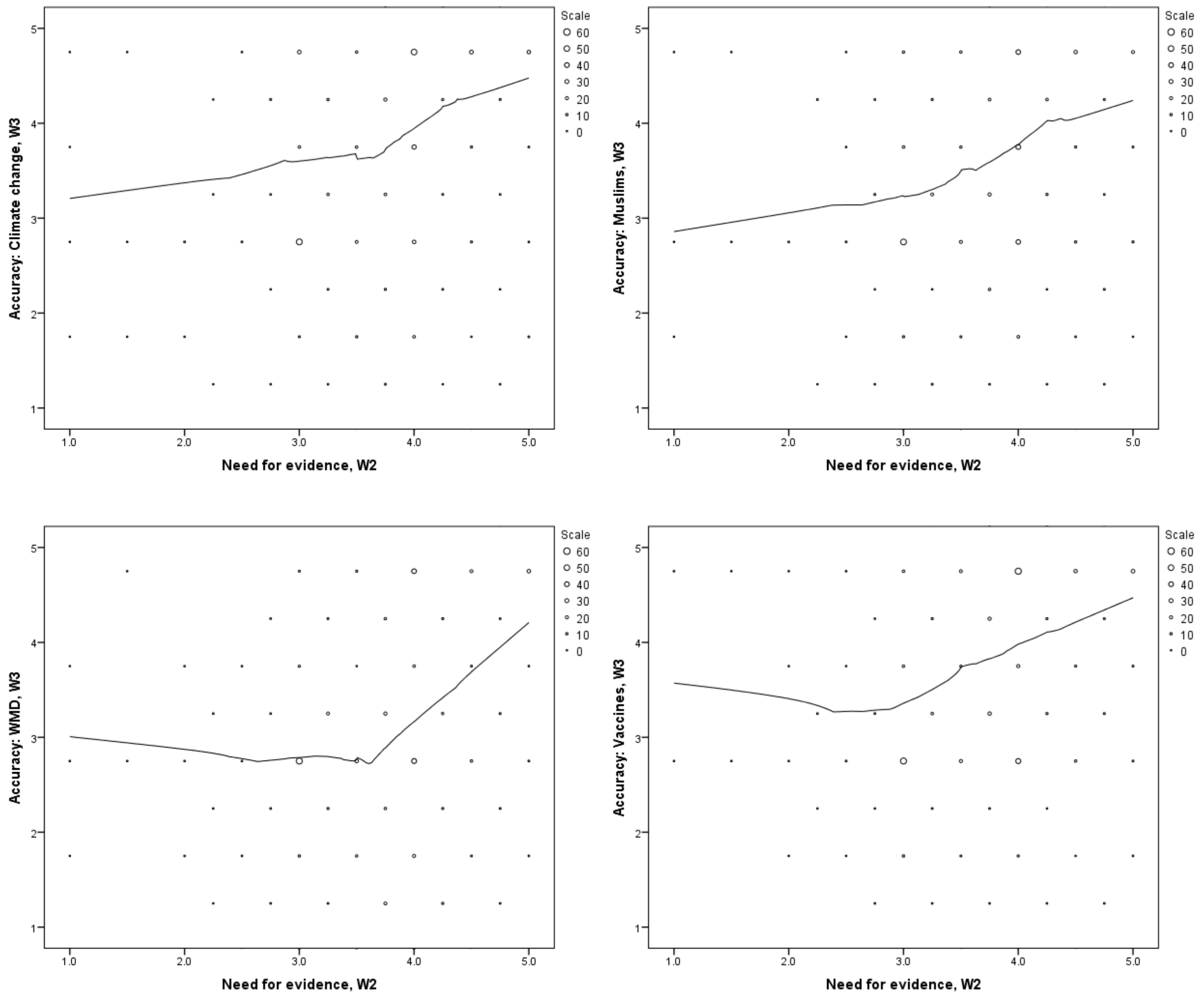

Supplement: S4 Fig — (PDF) [file pone.0184733.s009.pdf]
